# New opportunities and challenges for conservation evidence synthesis from advances in natural language processing

**DOI:** 10.1111/cobi.14464

**Published:** 2025-04-01

**Authors:** Charlotte H. Chang, Susan C. Cook‐Patton, James T. Erbaugh, Luci Lu, Yuta J. Masuda, István Molnár, Dávid Papp, Brian E. Robinson

**Affiliations:** ^1^ Department of Biology and Environmental Analysis Program Pomona College Claremont California USA; ^2^ The Nature Conservancy Arlington Virginia USA; ^3^ Department of Environmental Studies Dartmouth College Hanover New Hampshire USA; ^4^ Jornada Experimental Range New Mexico State University Las Cruces New Mexico USA; ^5^ Paul G. Allen Family Foundation Seattle Washington USA; ^6^ Lexunit Zrt Budapest Hungary; ^7^ Department of Telecommunications and Media Informatics Budapest University of Technology and Economics Budapest Hungary; ^8^ Department of Geography McGill University Montreal Quebec Canada

**Keywords:** conservation social science, evidence synthesis, language models, machine learning, natural language processing, aprendizaje automático, ciencias sociales de la conservación, modelos lingüísticos, procesamiento lingüístico natural, síntesis de evidencias

## Abstract

Addressing global environmental conservation problems requires rapidly translating natural and conservation social science evidence to policy‐relevant information. Yet, exponential increases in scientific production combined with disciplinary differences in reporting research make interdisciplinary evidence syntheses especially challenging. Ongoing developments in natural language processing (NLP), such as large language models, machine learning (ML), and data mining, hold the promise of accelerating cross‐disciplinary evidence syntheses and primary research. The evolution of ML, NLP, and artificial intelligence (AI) systems in computational science research provides new approaches to accelerate all stages of evidence synthesis in conservation social science. To show how ML, language processing, and AI can help automate and scale evidence syntheses in conservation social science, we describe methods that can automate querying the literature, process large and unstructured bodies of textual evidence, and extract parameters of interest from scientific studies. Automation can translate to other research agendas in conservation social science by categorizing and labeling data at scale, yet there are major unanswered questions about how to use hybrid AI‐expert systems ethically and effectively in conservation.

## Abstract (es)

Nuevas oportunidades y retos para la síntesis de evidencias de la conservación a partir de los avances en el procesamiento lingüístico natural

**Resumen:**

Abordar los problemas mundiales de conservación del medio ambiente exige traducir rápidamente los datos de las ciencias naturales y sociales de la conservación en información relevante para las políticas. Sin embargo, el aumento exponencial de la producción científica, combinado con las diferencias disciplinarias al momento de informar sobre la investigación, dificulta importantemente la síntesis interdisciplinaria de datos. Los avances actuales en el procesamiento lingüístico natural, como los grandes modelos lingüísticos, el aprendizaje automático y la minería de datos, prometen acelerar la síntesis interdisciplinaria de datos y la investigación primaria. La evolución de los sistemas de aprendizaje automático, procesamiento lingüístico natural e inteligencia artificial en la investigación científica computacional ofrecen nuevos enfoques para acelerar todas las etapas de la síntesis de evidencias en las ciencias sociales de la conservación. Para mostrar cómo el aprendizaje automático, el procesamiento del lenguaje y la IA pueden ayudar a automatizar y ampliar las síntesis de pruebas en las ciencias sociales de la conservación, describimos métodos que pueden automatizar la consulta de la bibliografía, el procesamiento de grandes volúmenes de pruebas textuales no estructuradas y la extracción de parámetros de interés de los estudios científicos. La automatización puede aplicarse a otros programas de investigación en las ciencias sociales de la conservación mediante la categorización y el etiquetado de datos a gran escala, pero aún quedan muchas preguntas por responder sobre cómo utilizar sistemas híbridos de IA y expertos de forma ética y eficaz en la conservación.

## INTRODUCTION

The world is facing extreme challenges, including human‐driven biodiversity loss (Díaz & Malhi, 2022), climate change (IPCC, 2018), and rising human needs for ecosystem services (Dasgupta, 2021; Pörtner et al., 2021). Addressing these issues will require integrating the natural and social sciences to find solutions to these multifaceted environmental and sociopolitical problems (Bennett et al., 2017; Dasgupta, 2021; Pörtner et al., 2023). Evidence synthesis is a crucial tool for bringing forth and compiling the best evidence across multiple fields, and several recent efforts have showcased how such syntheses can translate research into evidence‐based policy and practice (e.g., Cheng et al., 2019; McKinnon et al., 2016; Rosenstock et al., 2019).

Despite the importance of integrating scientific disciplines to identify and develop solutions to conservation challenges, several barriers prevent pooling diverse literatures for interdisciplinary understanding: the wide range of literature, inefficiencies related to text summarization, and errors or biases in data evaluation and coding. First, scientific evidence is dispersed across a range of peer‐reviewed outlets and disciplines. To review this dispersed evidence base, traditional synthesis methods require enormous logistical, financial, and community‐wide efforts. Second, numerous disciplines with diverse methodologies investigate relationships between people and nature. Ensuring consistency in coding evidence between people (interrater reliability) and over time (intrarater reliability), as well as addressing issues such as reviewer fatigue, can be a major challenge (DiMaggio, 2015; O'Mara‐Eves et al., 2015). Text data—from manuscripts or digital platforms—may be difficult to evaluate efficiently and consistently, particularly at large scales (Edelmann et al., 2020; Gentzkow et al., 2019). Finally, as the number of relevant articles increases over time (Callaghan et al., 2021; Sietsma et al., 2024; Thomas et al., 2017), the time and effort needed to conduct an evidence synthesis also increase. For example, a global synthesis effort focused on natural forest regrowth took 3 years and hundreds of hours of manual labor (Cook‐Patton et al., 2020). When published, the underlying database representing the most current synthesis was 3 years out of date and only covered priority areas for carbon accumulation.

Several recent advances in machine learning (ML) and natural language processing (NLP) provide new opportunities for synthesizing conservation social science and natural science. For example, conservation social scientists have applied NLP to news, social, and other media to examine public views of biodiversity or assess polarization in climate change discourse (Chang et al., 2022a, 2022b; Correia et al., 2021; Falkenberg et al., 2022; Giebink et al., 2024). These methods have utility in evidence synthesis (Farrell et al., 2022, 2024; Sietsma et al., 2024; Westgate et al., 2015), but potential users still struggle to understand the strengths and limitations of these tools (e.g., language bias, reliability). We sought to provide an overview of the emergence, spread, and advancement of ML and NLP in evidence synthesis. We also considered how such tools have become central to rapid and rigorous reviews.

## OVERVIEW OF EVIDENCE SYNTHESIS

**FIGURE 1:**
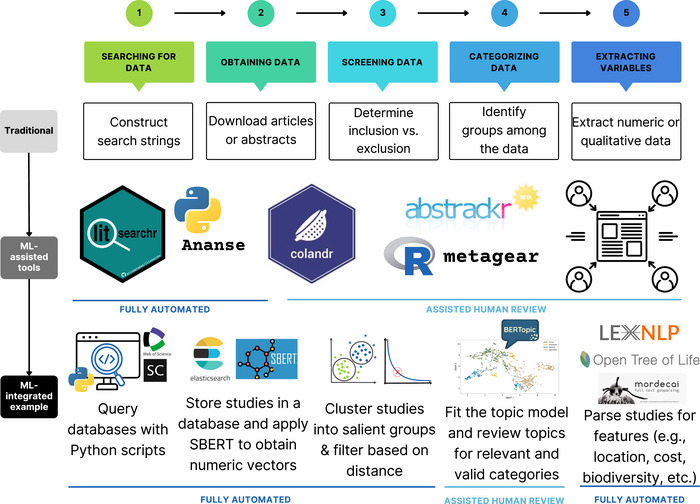
Traditional evidence synthesis tasks, examples of machine learning (ML)‐assisted tools (Table 1), and how ML and natural language process@#@ing (NLP) can be more deeply integrated throughout an evidence synthesis process (Table 2).

Systematic evidence syntheses often include developing search strings, screening and deduplicating search results, and coding for key variables of interest (Figure 1) (Grant & Booth, 2009; Khan et al., 2003; Lunny et al., 2017). Research teams focus on published research (e.g., Berrang‐Ford, Siders, et al., 2021; Callaghan et al., 2021; McKinnon et al., 2016) or additionally include unpublished data (e.g., IPBES, 2018; Porciello et al., 2020). Systematic reviews of peer‐reviewed publications are more common due to their prominence, replicability, and relatively complete indexing (Foo et al., 2021; Gusenbauer & Haddaway, 2020; van Driel et al., 2009).

ML or artificial intelligence (AI) tools can accelerate or automate every step in evidence synthesis (Figure 1; Tables 1 & 2), offering value when manual review and synthesis are insufficient and intractable. Even with teams of hundreds of experts collectively contributing thousands of person years of review effort, global efforts relying exclusively on manual review could only process 14,000 (IPCC, 2021) to 15,000 publications (IPBES, 2019). Arguably, the scale and state of global conservation problems now require much more rapid evidence synthesis. Aligning decision‐making with comprehensive, up‐to‐date information requires leveraging ML to accelerate identifying the best available scientific evidence base. ML‐ or AI‐assisted approaches could reduce bias and increase reproducibility relative to teams of human coders. Large language models (LLMs) hold particularly high promise. For instance, Callaghan et al. (2021) trained a relevance classifier on 2000 abstracts to predict whether >600,000 abstracts contained information on climate impacts. We outlined advances to date along each step of an evidence synthesis effort (Figure 1) that enable more rapid and robust data integration.

**TABLE 1:** Descriptions of different software packages that can support automated evidence syntheses in conservation social science.

Category	Tool	Purpose	Citation
Machine‐learning‐assisted tools	litsearchR	Determine search terms based on text mining and keyword co‐occurrence	Grames et al., 2019
	Ananse	Implementation of litsearchR in Python	Kwabena et al., 2023
	colandr	Semiautomated, human‐in‐the‐loop platform to screen abstracts for relevance	Cheng et al., 2018
	abstrackr	Semiautomated platform to screen abstracts for relevance	Gates et al., 2018; Wallace et al., 2012
	metagear	Tools to help teams of reviewers screen and process abstracts for relevance and other objectives	Lajeunesse, 2016
Machine‐learning‐integrated example	SBERT	Access and use transformer language models	Devlin et al., 2019; Reimers & Guryevich, 2019
	BERTopic	Perform topic modeling with transformer model input (contextual embeddings)	Grootendorst, 2022
	LexNLP	Structured information extraction for legal and financial documents	Bommarito et al., 2021
	Open Tree of Life	Comprehensive taxonomic information across the tree of life	Rees & Cranston, 2017; OpenTreeofLife et al., 2021
	Mordecai	Detect locations in text data	Halterman, 2017

**TABLE 2:** Resources for learning more about text analysis methods or replication code accompanying machine learning integrated syntheses.

Source	Focus	Link	Type
Chang et al., 2025	Natural climate solutions well‐being impacts	https://doi.org/10.5281/zenodo.14206056	Code repository
Jackson et al., 2022	Overview of text analysis for psychology	https://osf.io/hvcg3/	Code repository
Callaghan et al., 2021	Climate change impacts	https://doi.org/10.5281/ZENODO.5327409	Code repository
Berrang‐Ford, Sietsma, et al., 2021	Connections between climate and health	https://zenodo.org/records/4322697	Code repository

## ACCELERATING THE IDENTIFICATION OF RELEVANT ARTICLES

Developing search strings for academic databases or search engines is a foundational step for evidence syntheses and can itself be an involved process (Cooke et al., 2012; Villanueva et al., 2001). Keyword searches are often designed to capture the universe of potentially relevant publications and so may return orders of magnitude more articles than are actually relevant. Researchers may adjust keywords to achieve a manageable number of results, which can exclude relevant literature from review. New methods leverage NLP and network science to automate this process, producing more refined search strings (Grames et al., 2019; Kwabena et al., 2023; Figure 1; Table 1). Researchers then compile and screen articles for relevance and duplication. ML algorithms have assisted with screening for nearly a decade (O'Mara et al., 2015). Platforms such as abstrackr (Gates et al., 2018; Wallace et al., 2012), metagear (Lajeunesse, 2016), and colandr (Cheng et al., 2018) use human coding of a subset of abstracts and keywords to probabilistically evaluate the relevance of additional abstracts. These platforms use NLP to identify sentences or word clusters common among articles deemed to be relevant and assess if unscreened articles contain similar text (Cheng et al., 2018). As more articles are screened, the algorithm's accuracy improves (Tsou et al., 2020). Such a human‐in‐the‐loop process, where ML assists with article screening and coding that are checked by experts for validity, can balance cost efficiency and timeliness with consistency (O'Connor et al., 2019; Thomas et al., 2017). Such approaches have been used in global evidence synthesis projects.

For example, Berrang‐Ford, Siders, et al. (2021) used NLP to screen 48,000 articles and an expert team of 126 researchers who collectively coded 1682 articles for evidence on climate adaptation. However, in Berrang‐Ford, Siders, et al.’s (2021) review, attributes such as the regional focus of each included study (*n* = 2032) were manually extracted by teams of experts. Evidence syntheses featuring greater automation have used ML and AI systems to go beyond predicting article relevance to predicting relevant categories or features in and across studies. For example, researchers have predicted labels for individual studies focused on global development evidence for agriculture and food security (Edwards et al., 2024), climate change impacts (Callaghan et al., 2021), or natural climate solutions impacts to human and environmental well‐being (Chang et al., 2025).

## AUTOMATING DATA QUERIES IN EVIDENCE SYNTHESIS

AI systems can automate additional steps in evidence synthesis workflows, such as querying and downloading studies (e.g., Berrang‐Ford, Siders, et al., 2021; Callaghan et al., 2021; Edwards et al., 2024; Sietsma et al., 2024). For example, scripts and application programming interfaces (APIs) have been used to query and retrieve scientific abstracts and other relevant publication information from Scopus and the Web of Science in a more automated fashion (Callaghan et al., 2021; Chang et al., 2025). In addition to saving time, this approach is more transparent and replicable, which avoids errors that can emerge in manual data querying (e.g., data entry errors, deviations from protocol). Further, manual querying may not be feasible for some syntheses. Our process culminated in over 2 million abstracts—a volume that would be difficult, and perhaps impossible, to manually download.

There are some advantages of manual data curation that are not typically integrated into ML‐assisted workflows. For instance, experts may be interested in expanding their evidence base by following a citation trail from initially selected studies. However, automated querying approaches can emulate these tasks through careful design. For example, one might obtain citation networks from scholarly databases and use supervised classification to predict which abstracts may correspond to areas that would benefit from additional samples (Ammar et al., 2018; Priem et al., 2022).

Moving beyond abstract‐level data to automatically accessing the full text of scientific articles is at the cutting edge and has been facilitated by open science mandates and platforms, such as PubMed Central (Farrell et al., 2022). Open‐source tools can extract text, tables, and figures from PDFs or parse the full text of articles delivered in structured formats such as HTML (Liu & McKie, 2024; Yang et al., 2019). To date, however, we are not aware of examples of extensive automation of such full‐text features to accelerate evidence synthesis in conservation. The diversity of disciplines, methodologies, and results (e.g., tables and figures presenting statistical analyses, qualitative analyses and results, etc.) presents a unique challenge for adopting these methods in conservation and beyond.

## EVOLUTION OF TEXT ANALYSIS TIME TO PROCESS BODIES OF EVIDENCE

Key to any evidence synthesis project is acquiring information and extracting data from relevant articles, such as cataloging themes or topics across studies. We considered how advances in text analysis approaches through time have enabled different types of analyses. With manual reviews as a starting point, data extraction followed a coding template to record information consistently (Grant & Booth, 2009; Khan et al., 2003; Lunny et al., 2017). Research in the social sciences and conservation social science shows how manual coding can be supplanted or augmented by NLP (Ash & Hansen, 2023; Grimmer et al., 2022). In some of the earliest advances in computational social science, relatively simple statistical NLP methods, such as frequency‐based n‐grams that identify a sequence of adjacent terms, were used. Although simple, these earliest NLP methods offered major new insights. For example, Michel et al. (2011) provided a watershed advance for quantitatively researching human culture by assessing n‐gram patterns across time in the Google Books corpus. Anderson et al. (2021) used a similar n‐gram approach to document shifts in ecological research across 3 decades.

Other researchers have used probabilistic, unsupervised language models, most notably topic models (Blei, 2012; Boyd‐Graber et al., 2017; Grimmer et al. 2022). A topic model is an unsupervised clustering model that discovers themes across documents based on the assumptions that documents are a mixture of themes (or topics) and that different topics tend to be associated with different terms—for instance, zoonotic disease is described with terms distinct from those for habitat loss. In conservation social science, topic modeling has been used to track trends in climate disinformation (Farrell, 2016) or environmental discourse on social media (Chang et al., 2022a, 2022b). NLP in general and topic modeling in particular have been used relatively rarely in evidence syntheses. However, in a few literature reviews, topic models were used to document shifts in conservation science and common pool resource research (Lambert et al., 2021; Westgate et al., 2015). Topic models are appropriate when researchers do not have strong prior knowledge on which categories clearly exist in a data set. Berrang‐Ford, Sietsma, et al. (2021) used topic modeling to induct categories in a global evidence review focused on climate hazards and public health.

The outputs of probabilistic language models or simpler keyword‐ or n‐gram‐based outputs can be further augmented. For example, researchers could use sentiment analysis to quantify valence (e.g., positive to negative labels), emotions (e.g., fear vs. disgust vs. hope), or opinions in text (Mohammad, 2016). Van Houtan et al. (2020) used a sentiment analysis of keywords in species reintroduction studies to examine drivers of successes versus failures. These earlier studies processed text data with relatively simple bag‐of‐words vectorization, where each span of text would be converted to a list of distinct words. Such text vectorization, however, loses the rich meaning conveyed in the order of words and the semantic structure of human language.

NLP can extract additional features in scientific articles or text data, such as study locations, species studied, or financial measures (Farrell et al., 2022; Figure 1). Named entity recognition (NER) is an NLP task that evaluates how well models can detect and correctly classify entities. One example of an NER task is differentiating Lima, Peru, as a place name from lima bean as a food item. Detecting location from text data often relies on NER to identify place names, which are then resolved to geographic coordinates (Halterman, 2017).

Recent advances in NLP, such as transformer language models, are at the vanguard of evidence synthesis and are particularly relevant for conservation social science due to their ability to distill insights from much larger bodies of evidence while capturing more nuanced meaning and context than bag‐of‐words approaches (Ash & Hansen, 2023; Burnham, 2024). Transformer language models are deep neural network models designed to capture associations between spans of text and predict which portions of the text are important (Vaswani et al., 2017). As a result, transformer language models, such as Google's Bidirectional Encoder Representations from Transformers model (BERT) (Devlin et al., 2019), can better represent subtle variations in meaning in different pieces of text. The BERT model has been used as an input for supervised models to categorize evidence for climate impacts and adaptation (Callaghan et al., 2021) (Table 2), food systems (Porciello et al., 2020), and global development and food security (Edwards et al., 2024).

Transformer models, such as BERT, can also be used in unsupervised topic models. For example, Chang et al. (2025) aimed to discover what combinations of natural climate solutions (NCS) pathways and impacts to human and environmental well‐being existed in a large body of scientific evidence (Table 2). NCS refers to a set of preexisting management practices grouped in new categories. For instance, extended timber rotation is one practice contained in the improved forest management NCS pathway (Griscom et al., 2017). Therefore, Chang et al. (2025) were uncertain which pathway and impact combinations would even exist as valid categories. A topic modeling procedure that included outputs from the BERT model provided results that were robustly sampled across a large set of abstracts. These results were then mapped to combinations of NCS pathways and impacts.

Review teams have consistently reported significant time savings from ML‐assisted or automated evidence synthesis. Simple abstract screening tools have reduced workload by 35–99% at various stages (Gates et al., 2019). Edwards et al. (2024) found that their automated system cut human screening effort by 55%. Chang et al. (2025) reported that manually reviewing their initial sample of over 2 million abstracts would have been infeasible, even with a large expert team dedicating thousands of hours. Instead, by reviewing the outputs of the BERT topic model (Figure 1; Table 1), which included, for example, highly relevant abstracts for each cluster, their core team of 5 data coders managed the task in just tens of hours per person (Chang et al., 2025).

However, ML‐supported or automated systematic reviews and meta‐analyses also introduce challenges. These challenges include selecting ML models (Marshall & Wallace, 2019; Thomas et al., 2017) and ensuring that ML‐assisted findings are valid (Qureshi et al., 2023). Additionally, many ML algorithms require practitioners to make choices about hyperparameters or model settings that are not directly estimated from data. For instance, in a categorical prediction model that always returns 5 categories, the number of categories output is itself a hyperparameter. As an example in the context of topic modeling, Farrell (2016) had to determine the number of topics for categorizing climate disinformation generated by fossil fuel companies. Similarly, to categorize environmental discourse with a topic model, Chang et al. (2022b) had to consider hyperparameters such as the numbers of topics and ways to pool social media posts.

In Chang et al. (2025), one critical hyperparameter was the number of clusters used to categorize NCS co‐impacts. That study used Bayesian optimization to identify the hyperparameter combinations that maximized the coherence of the resulting topics (Chang et al. 2025; Snoek et al., 2012). Coherence has been a standard way to examine the parsimony and fit of topic model results (Mimno et al., 2011), and a more coherent model will have topics that are highly distinct from other topics (Grimmer et al., 2022). Chang et al. (2025) noted that Bayesian optimization was necessary because, given the 5.6 million possible hyperparameter combinations, one would otherwise require almost 320 years to perform a grid search over all possible combinations. Bayesian hyperparameter optimization methods balance feasibility with rigor. Hyperparameters and other model design choices are also relevant for supervised classification models. For example, practitioners must make choices ranging from the division between training and test data to the minimum number of examples that can constitute a valid category (Grimmer et al., 2022; Sietsma et al., 2024).

Free resources are available for practitioners seeking to learn more. Some literature reviews offer an overview of ML and AI integrated methods in social science (e.g., Jackson et al., 2022) (Table 2), and there are NLP courses and tutorials online (e.g., Climate Change AI, Summer Institutes in Computational Social Science, and the open‐source platform Hugging Face). However, the field is changing rapidly, and data sources or code to implement certain analyses can become deprecated in unpredictable ways.

## FUTURE DIRECTIONS FOR NLP, ML, AND AI IN CONSERVATION SOCIAL SCIENCE

Generative pretrained transformer (GPT) LLMs offer new possibilities for conservation social science evidence synthesis because such models can excel at identifying relatively nuanced, qualitative attributes from text corpora (Farrell et al., 2024). Such LLMs, also known as foundation models, because they have been trained on a broad range of data, have shown state‐of‐the‐art performance across a wide range of downstream uses (Kojima et al., 2022). Henceforth, we use the term *LLM* to refer to GPT types of models. An LLM can automate tasks such as content analysis (Chew et al., 2023), a common task in qualitative studies. Scheepens et al. (2024) demonstrate the use of OpenAI's GPT‐4 LLM to automatically extract hierarchical and taxonomic information on pests and pest controllers, such as natural enemies or parasitoids, from the ecological literature.

Research in the social sciences provides new examples, seemingly by the week, of how LLMs can augment the efforts of human coders in data labeling. LLMs have shown high levels of performance for categorizing political opinions (Burnham, 2024); psychological or political science labels, such as disinformation (Ziems et al., 2024); and affective and cognitive attributes in text, such as implicit bias (Demszky et al., 2023). Moreover, some LLMs, particularly closed‐source LLMs, can generalize across languages beyond English. Smaller, open‐source LLMs are also increasingly being trained on multilingual data sets, offering exciting possibilities for examining non‐English language evidence with open‐source models (Grattafiori et al., 2024). Some LLMs may outperform human coders for NLP tasks, such as sentiment analysis or moral values coding, even for languages with little to no labeled training data (e.g., Turkish, Kinyarwanda, or Bahasa Indonesia) (Rathje et al., 2024). Most remarkable is that these LLMs can achieve best‐in‐class outcomes without any custom training data. In some cases, even with zero‐shot predictions, LLMs have surpassed specialized deep learning models by a substantial margin. Taranukhin et al. (2024) documented that zero‐shot LLM climate opinion predictions have an F_1_ score (the harmonic mean of precision and recall) 28.4 points higher than previous, specialized models.

Use of LLMs to perform qualitative data coding in conservation science is still relatively limited (Farrell et al., 2024). Yet, the power and performance of these models across a variety of domains offer exciting possibilities for conservation social science research and synthesis. For example, LLMs could assist evidence synthesis teams in coding abstracts or primary data to answer questions, such as what services and values nature provides to people; determine the impacts of conservation management on local communities; and identify how much attention research topics or management approaches have received. Arguably, systems that automate data querying, processing, and categorization with ML and NLP could lead to living evidence syntheses that generate updates of new evidence on a regularly scheduled basis (Farrell et al., 2022; Sietsma et al., 2024; Thomas et al., 2017).

Large closed‐ and smaller open‐sourced LLMs have exceeded nonexpert human coders in accuracy at a fraction of the cost (Alizadeh et al., 2025). These results suggest exciting horizons for hybrid LLM‐expert systems for large‐scale evidence reviews for which complete manual review is infeasible. Nevertheless, there are still substantive methodological questions without one‐size‐fits‐all solutions, such as the most appropriate way to engineer prompts for zero‐ or few‐shot LLM predictions (Farrell et al., 2024; Qureshi et al., 2023; Scheepens et al., 2024). In contrast to traditional manual data coding and evidence synthesis, research on bias in ML for environmental evidence synthesis remains limited (Farrell et al., 2024; Sietsma et al., 2024). We posit that interdisciplinary conservation social science teams could make exciting contributions in the domain of ethics and effective use. New guidance that builds on existing frameworks, such as the CARE principles for Indigenous data governance (Carroll et al., 2020; Jennings et al., 2023) and FAIR data management principles (Wilkinson et al., 2016), for LLM‐driven analyses is critically needed.

Transparency may help in this endeavor. Explainable AI (XAI) aims to make AI decisions more transparent through generated explanations (Adadi & Berrada, 2018; Phillips et al., 2020), a concept rooted in rule‐based systems from nearly 50 years ago (Scott et al., 1977; Xu et al., 2019). Although XAI enhances transparency, curbs ethical risks, and accelerates discovery, rigorous validation across diverse data, tasks, and models is still crucial (Nauta et al., 2023). Careful scoping guided by interdisciplinary expertise, cocreating AI systems with conservation and community partners, and accounting for maintenance and deployment upfront will make AI systems more positively efficacious (Gebru & Torres, 2024; Moorosi et al., 2023).

In general, human review is essential because LLMs can fail at coding data in unpredictable ways, particularly for topics or attributes that are rare, are esoteric, involve complex logical reasoning, or require difficult trade‐offs with no single correct decision (Kristensen‐McLachlan et al., 2023; Zhu et al., 2023). Alizadeh et al. (2025) relied on data annotated by trained individuals using a structured codebook to ascertain LLM and crowdworker coding accuracy. Consider, for example, coding the statement, *The shift to electric vehicles was seen as a key climate action, though some pointed to the environmental costs of battery production*, for a positive, negative, or neutral opinion toward decarbonization via electrification. One could conceivably argue for any of those possible classes; thus, any AI system is unlikely to then come to a universally accepted decision. Reflecting on collaborations between social and computer scientists, DiMaggio (2015) wryly concluded that although computer scientists trust human judgment as a gold standard, social scientists, wary of human biases, hope algorithms can outperform flawed and inconsistent human reasoning, only to find that both can fall short in similar ways.

## DISCUSSION

NLP and ML techniques are evolving rapidly. These new techniques promise to disrupt expectations related to the resources that evidence syntheses require and the scale and speed at which they can be conducted. For example, the latest advances in LLMs can accelerate automation of middle‐ and end‐of‐process steps in evidence collation and synthesis (e.g., filtering, data extraction, and analysis) (Farrell et al., 2024) (Figure 1; Marshall & Wallace, 2019; Thomas et al., 2017). For conservation scientists interested in topics that are not confined to a specific discipline or literature, such as climate change resilience, land tenure security, and environmental justice, existing and new NLP tools will continue to accelerate the synthesis of greater evidence volume (Farrell et al., 2022; Sietsma et al., 2024). This reflects a broader need for advancing sustainability science: the integration and synthesis of information with a focus on systems rather than disciplines.

Several areas require further research and debate for the rigorous application of NLP to evidence syntheses across conservation. ML and NLP do not provide a silver bullet for all evidence synthesis or primary research analysis needs (Marshall & Wallace, 2019; Thomas et al., 2017). At present, they are most useful when synthesis focuses on a broad topic with vast evidence. Assessing when and to what extent human review is necessary remains important for advancing ML synthesis methods. Given the emerging state of algorithms applicable at different review process steps (Figure 1; Table 1), expert guidance and review are essential (O'Connor et al., 2019; O'Mara‐Eves et al., 2015; Qureshi et al., 2023).

Future advances in ML, NLP, and data extraction offer promise for enhancing the scope of automation. Increased ability for LLMs to take in large amounts of text at once (longer context windows), for example, makes it increasingly possible to process a published article in its entirety to more fully determine relevance for inclusion or to extract richer insights. Ongoing and future developments may make it possible to straightforwardly and robustly extract text versus data in tables and figures (Liu & McKie, 2024; Yang et al., 2019). However, setting and meeting uniform standards for data archiving and results presentation in papers would help synthesis science flourish with or without advanced ML tools (Fidler et al., 2017; Michener, 2015; Reichman et al., 2011; Sietsma et al., 2024).

Although NLP can integrate human checks of data, there are currently no standards or guidelines for determining acceptable thresholds and cutoffs for key decision points, which may lead to inconsistent use and results (O'Connor et al., 2019). In general, evidence syntheses must contend with uncertainty, whether workflows are automated or manual. NLP can introduce uncertainty when models use probabilistic determination, such as decisions on including versus excluding evidence. In contrast, manual syntheses include a comprehensive review of the entire sample set but may contain inconsistencies among human reviewers, uncertainties regarding inclusion criteria or coding, and human errors. Such mistakes can be mitigated but not eliminated by, for example, double coding. The assumption of human coding primacy has been challenged in interesting and difficult‐to‐anticipate ways (DiMaggio, 2015; Ziems et al., 2024). Thus, an intriguing use case for ML‐assisted systems is correcting mislabeled data generated by human coders (O'Mara‐Eves et al., 2015). As new tools leveraging cutting‐edge NLP techniques emerge, future research should revisit assumptions in workflow design (O'Connor et al., 2019).

Although there are guidelines for traditional evidence syntheses (Haddaway et al., 2018; Page et al., 2021), thus far, there are no equivalent guidelines for AI‐assisted syntheses in conservation and the environment. As a starting point, Beller et al. (2018) and O'Connor et al. (2019) discuss best practices for automated systematic reviews. Groups such as the Campbell Collaboration (2023) have also proposed future directions for standards of NLP and other ML techniques for evidence synthesis.

Conservation scientists eager to employ NLP for evidence synthesis must also grapple with doing so equitably. Existing NLP tools have an English language bias (Bang et al., 2023; Blevins & Zettlemoyer, 2022; Huang et al., 2023). Despite the surprising and strong performance of closed‐source LLMs in multilingual text classification (Rathje et al., 2024), AI researchers caution that multilingual data sets, especially for the lowest‐resource languages, may instead contain nonsense or machine‐generated text rather than human writing (Hadgu et al., 2023). Overcoming these inequities requires a concerted effort to develop LLM training and instruction‐tuning data for non‐English languages.

There are substantive ethical questions about using AI for any social endeavor (Gebru & Torres, 2024), one of which revolves around the energy and environmental footprint of the largest models (Bender et al., 2021; Luccioni et al., 2024). Given that the energy and environmental costs of training LLMs have already been incurred, some would argue for applying existing models to conservation use cases. However, generative AI models demand much more energy and water than previous ML models; estimates indicate that one generative AI search request could demand 10 times as much energy as a conventional search (de Vries, 2023; Li et al., 2023; Luccioni et al., 2024). Moreover, although LLMs can provide positive benefits, there are also risks that these models reproduce social harms, such as biases in the training data that preclude marginalized groups or languages from inclusion (Bender et al., 2021; Hadgu et al., 2023) and privacy violations (Staab et al., 2023). Researchers are beginning to grapple with how to anticipate and combat potential harms in generative AI, calling for greater public accountability, fairness, and transparency (Gebru & Torres, 2024; Harrer, 2023).

Finally, as NLP methods advance, there must be an emphasis on transparency and replicability. Code and data should be publicly available (O'Connor et al., 2019). We should be able to call on time‐stamped versions of LLMs so that results can be replicable; indeed, this is arguably an advantage for open‐source LLMs (Abdurahman et al., 2024). Synthesis teams should fully document the tools they develop, flag where possible biases may emerge, and report uncertainty when appropriate (Sietsma et al., 2024; Thomas et al., 2017). This is especially important given that NLP analyses are often black‐box processes and results are sensitive to choices, such as prompt engineering (Khattab et al., 2023; Kojima et al., 2022).

We outlined how emerging approaches to evidence synthesis can transform environmental, conservation, and sustainability science. Although we provided up‐to‐date examples, we emphasize that these methods are relatively new and poised to develop rapidly. Critical to the success of this burgeoning field will be developing a high‐level process that can ensure the reliability and validity of ML‐aided robust synthesis efforts. Equally important is the development of ethical frameworks to ensure that ML tools are applied in ways that reflect the diverse needs of all stakeholders. The potential for ML to enhance learning is vast, but it must be complemented by use‐oriented design, stakeholder engagement, and multidisciplinary collaboration to guide ML‐assisted efforts to the best global outcomes.
